# Itch in Allergic Contact Dermatitis

**DOI:** 10.3389/falgy.2021.702488

**Published:** 2021-10-04

**Authors:** Julien Lambert

**Affiliations:** ^1^Antwerp University Hospital, Antwerp, Belgium; ^2^University of Antwerp, Antwerp, Belgium

**Keywords:** itch, pruritus, contact dermatitis, contact allergy, mast cells

## Abstract

Contact dermatitis is a continuous growing environmental and occupational health problem. It results in high costs for health care systems and the economy due to productivity loss. Moreover, it has a huge impact on the quality of life of patients. The immune response to contact allergy is very complex and not totally elucidated. Recently unique pathways preferentially activated by different allergens were identified. As for a lot of chronic itch disorders, antihistamines are ineffective for allergic contact dermatitis, suggesting a non-histaminergic itch. The precise mechanisms that underlie the development of itch in ACD remain poorly defined. This short review addresses the most recent insights in pruritus in ACD, opening perspectives for future therapies.

## Introduction

Contact dermatitis is a common reason of consultation: skin problems provoked by contact with chemicals is a growing environmental and occupational health problem (1).

We distinguish 2 types of contact dermatitis, taking into consideration the pathophysiological mechanism, namely irritant contact dermatitis (ICD) and allergic contact dermatitis (ACD).

Irritant contact dermatitis represents 80% of all contact dermatitis cases. It can occur after a single exposure to an irritant or toxic substance, inducing skin damage due to a direct and local cytotoxic effect. Clinical lesions can vary from erythema to a vesicular reaction, or even a caustic burn with necrosis. In case of low grade irritants it will take much longer to observe clinical manifestations. This chronic type of ICD can be observed after accumulative and repetitive exposure to irritative substances such as soap and detergents.

The other 20% of cases of contact dermatitis are due to ACD. ACD presents as an itchy eczematous reaction of the skin, occurring hours to days after contact with an allergen. In a chronic phase it presents as erythematous, scaly and lichenified lesions. ACD affects about 20% of the adult general population (2, 3). A recent study showed that 27% of the general population from 5 European countries had contact allergy (this means sensitization to at least one contact allergen of the European baseline series) (4). A large proportion of these individuals are at risk of developing ACD following exposure to these allergens. These figures reveal the importance of ACD as health problem for the society. For the individual patient ACD may be a very bothersome skin problem. Although the mainstay of ACD treatment, namely allergen avoidance, seems logical and rather easy it is in real life frequently difficult. The omnipresence of the allergen can make avoidance very difficult, exposure to very small amounts can be enough to trigger a reaction and not always can the culprit allergen be identified (5).

As itch is a dominant symptom in ACD with a huge impact on patient's quality of life, we would like to present a short overview of the current knowledge concerning the mechanisms responsible for itch in patients with ACD. Even if the precise mechanisms that underlie the development of itch in ACD remain poorly defined, every new step in understanding could lead to novel therapeutic approaches.

## Pathophysiology of ACD

ACD is a delayed type IV hypersensitivity reaction to a hapten or non-protein contact antigen. Its pathophysiology is characterized by 2 phases, which involve both innate and adaptive immune responses (1). First is the sensitization phase, also called the afferent or induction phase, referring to the first contact with the allergen, including the penetration of the stratum corneum and the development of effector T cells. During the second phase, the elicitation phase or effector or challenge phase the patient is re-exposed to the sensitizing allergen, resulting in clinical manifestations, normally observed within 24–72 h. Most of the scientific data on ACD have been obtained from the mouse model for ACD, the murine contact hypersensitivity model (CHS) (6). For these studies strong contact sensitizers were used, such as dinitrochlorobenzene (DNCB), trinitrochlorobenzene (TNCB), dinitrofluorobenzene (DNFB), squaric acid dibutylester (SADBE) and oxazolone, which are experimental allergens not present in our daily environment, in contrast to weak sensitizers clinically relevant to human ACD, such as nickel and fragrance. Also for a few of these clinically relevant sensitizers murine models were established, namely nickel and urushiol (the major allergen in poison ivy) (7, 8). Classically ACD is considered as a Th1 mediated disease or a mixed Th1/Th2 response, but over the last years experimental evidence revealed the role of Th17 and Th22 cytokines (9). The study of Dhingra et al. defined the common transcriptome of clinically relevant sensitizers in human skin and identified unique pathways preferentially activated by different allergens (9). Nickel exhibited potent induction of innate immunity and Th1/Th17 polarization while fragrance and to a lesser extent rubber demonstrated a strong Th2 bias some Th22 polarization and smaller Th1/Th17 contribution. Finally human poison ivy ACD seems to involve Th2 and Th17 type (10). Distinct immune polarizations to specific allergens have been observed not only in human ACD patients but also in animal models. The experimental allergens DNFB and TNCB for example exhibit mainly Th1-type immune responses in rodents, FITC and the urushiol model (mouse model of poison ivy ACD) on the contrary a Th2 biased immune response (10, 11). The oxazolone model exhibits a Th1/Th2 mixed immune response (10) (see Table 1).

**Table 1:** Distinct immune pathway activations by different allergens.

**Experimental allergens**	
Initrofluorobenzene (DNFB)	Th1
Trinitrochlorobenzene (TNCB)	Th1
Fluorescein isothiocynate (FITC)	Th2
Urushiol	Th2
Oxazolone	Th1/Th2
**Human allergens**	
Nickel	Th1/Th17 Th22 component
Fragrance	Th2/Th22 (weaker Th1/Th17 axis)
Poison ivy	Th2/Th17

## Additionally Recent Studies Suggest a Role for IL-23 and IL-25 in ACD

IL-23 is a pro inflammatory cytokine from the family of the IL-12, which plays a role in the development of Th17 cells. The Th2/Th22 immune response is the main factor in the development of atopic dermatitis but the Th1 immune response and the Th17/IL-23 signaling pathway could play a role in the transition to a chronic state (12, 13). IL-23 seems to be involved in the pathogenesis of ACD, however there are relatively few studies on IL-23. One of these showed in case of sensitization of ACD mice with oxazolone increased interferon γ-(IFN-γ), IL-17α and IL-23 levels and accumulation of CD8 tissue resident memory cells in the skin (14).

IL-25 (also called IL-17-E) is produced by epithelial cells and various immune cells. It can induce Th2 cell differentiation and activation suggesting its involvement in Th2-type immune responses.

A recent study investigated the contribution of IL-25 in a mouse model of CHS challenged with fluorescein isothiocyanate: the results showed that unexpectedly mast cell- and non-immune cell-derived IL-25 was important for hapten-specific Th17 cell - mediated rather than Th2 cell - mediated inflammation in the elicitation phase by enhancing Th17 related but not Th2-related, cytokines in the skin (15).

These observations demonstrate that ACD is not a single immunological process. There seems to be a mechanistical difference between allergens. This means also that animal models may not be representative of environmental cases of ACD. Moreover, these insights could have therapeutical consequences: targeted-therapy, taking into consideration sensitivity to a specific allergen could enhance efficiency, especially for difficult to treat cases not responding to the classical treatments.

Although ICD and ACD share similar clinical characteristics their immunological mechanisms are quite different. In the early stages, ICD and ACD may share cytokines associated with the activation of the innate immune system, but in ICD there is no involvement of antigen-/allergen–specific T cells and the adaptive immune system (6). Direct skin damage activates the innate immune response: keratinocytes release IL-1α, IL-1B, tumor necrosis factor α (TNF-α), granulocyte-macrophage colony stimulating factor (GM-CSF) and IL-8. These activate Langerhans cells, dermal dendritic cells and endothelial cells resulting in recruitment of neutrophils, lymphocytes, macrophages and mast cells to the site of keratinocyte damage (16).

## Non Histaminergic Itch in ACD

Itch is initiated when endogenous or exogenous pruritogens interact with itch receptors or pruriceptors (pruritus + receptor), which resides in the membrane of the nerve endings of primary afferent C fiber somatosensory neurons (17). There are three classes of receptors that can be activated by itch mediators namely G protein-coupled receptors (GPCR), toll-like receptors (TLR) and cytokine receptors (18). Exogenous environmental stimuli or endogenous molecular and cellular components interact in a direct or indirect manner with sensory neurons. A contact allergen is an example of an indirect stimulus: it can generate an allergic reaction leading to the release from immune cells, of itch mediators which then stimulate sensory neurons (18).

## Mast Cell-Mediated Itch in ACD

The most well defined form of itch is histamine-mediated or histaminergic itch. Mast cells are the key players via release of histamine which activates receptors present on itch-sensory neurons of the dorsal root ganglia (DRG). Mast cell activation results from antigen binding to IgE antibody and cross-linking of the high-affinity IgE receptor, (Fc ε RI). Antihistamines however, are ineffective for ACD like it is the case for a lot of chronic itch disorders such as atopic dermatitis (AD) (5). ACD is also an example of diseases characterized by persistent itch in contrast to transient itch and accompanying nociceptor responses. Less is known about the nociceptor activity during itch lasting for days or longer (19).

Mast cells are often found in close association with nerve fibers in peripheral tissues (20). Apparently, bidirectional communication between mast cells and nerves lead to itch, pain, and inflammation (21). It is only in recent papers that the molecular mechanisms concerning mast cell-nerve communication are highlighted. Recently, members of the Mas-related family of G-protein-coupled receptors (Mrgprs), namely Mrgprb2 in mice and MRGPRX2 in humans were discovered as mediators of mast cell activation (22, 23). They are activated by basic secretagogues, which include 48/80, the neuropeptide substance P (SP), pro-adrenomedullin peptide 9-20 (PAMP9-20), known to activate mast cells through a non-IgE mechanism. Recent studies showed that the mast cell degranulation through the activation of these receptors was different both spatially and temporally from FCεRI-mediated degranulation (22, 24). These observations launch the hypothesis that mast cell Mrgpr pathways may promote itch in an unique fashion, separate from classical IgE-mediated itch.

The recent study of Meixiong et al. delivers very interesting findings for the scope of this overview: distinct types of mast cell stimuli induce a differential release of mast cell mediators leading to the activation of distinct sets of sensory neurons and itch modalities (25). Mrgprb2–mediated mast cell activation led to secretion of tryptase β2 and low levels of serotonin. FCεRI-mediated mast cell activation led to the secretion of high levels of histamine and serotonin but not tryptase β2. Comparison of the functional outcome of these two types of mast cell degranulation leads to distinct types of itch namely histamine-dependent and histamine-independent itch. Interestingly, IgE-FCεRI-associated itch had also a non-histaminergic component resistant to histamine receptor antagonism and a minor component of Mrgprb2-associated itch may be histaminergic, as antihistamines slightly reduced the number of sensory neurons activated by PAMP9-20, ligand of Mrgprb2. These observations suggest some overlap between Mrgprb2-and FCεRI-mediated mast cell itch.

Additionally they observed that the distinct mast cell activation led to activation of distinct neuronal subtypes. Neurons excited by mast cell activation through PAMP9-20 (ligand of Mrgprb2) most consistently overlapped with Mrgpd+ itch neurons, while neurons excited by mast cell activation through anti-IgE overlapped with histamine-sensitive neurons. In itch sensory neurons, H1R expression and Mrgprd expression do not overlap (26), an indication that PAMP9-20 and anti-IgE activated different populations of sensory neurons (25). A possible explanation for this distinct sensory neuron activation between PAMP9-20 and anti IgE could be differential mast cell degranulation and release of compounds like tryptase β2

Finally Meixiong et al. studied the role of Mrgprb2 in ACD: itch behavior was significant reduced in Mrgprb2 -/- mice compared to wildtype (WT) mice for three classical models of ACD (using SADBE, oxazolone and DNCB). Total CD45+ immune-cell numbers were reduced in Mrgprb2-/- mice compared to WT mice in DNCB-treated mice, these findings are an indication Mrgpr signaling could play a role in immune cell recruitment in ACD. The authors also observed increased levels of PAMP1-20, an MRGPRX2 agonist as well as increased numbers of mast cells in the skin of ACD patients in comparison to healthy controls. All these data suggest a clear role of mrgprb2 (mice) and MRGPRX2 (human) in ACD itch. Also to note is that Mrgprb2-/- animals had a significant reduction in pruritus but still had some residual ACD itch. This could be explained by alternative mechanisms, such as IL-33 activation of neuronal ST2 and T cell release of IL-31 (7, 25, 27) (See below).

Analyzing the results of Meixiong et al. important questions may be put forward (21). Which mediator(s) activate pruriceptive neurons following Mrgprb2 activation; a possible candidate is tryptase-β2. Another question is whether mast cells and sensory neurons form a bidirectional positive feedback loop in itch and inflammation. Following activation sensory neurons release neuropeptides, including SP, a potent activator of MRGPRX2 and Mrgprb2.

## Cytokine and cytokine Receptors

Cytokines are a broad category of signaling molecules utilized by immune cells and keratinocytes for communication. It has been recognized in recent years that itch sensory neurons also express cytokine receptors and that cytokines may act as pruritogens (17, 28). Upon stimulation they are released by skin or immune cells and form a bridge of communication between the immune and nervous system. We present the actual knowledge about the cytokines involved in itch in ACD.

## IL-31

The role of IL-31 causing pruritus has been put forward, especially in atopic dermatitis, but also in other skin diseases as ACD. One of the prime distinguishing aspects of IL-31 is that it induces late onset pruritus, in contrary with the immediate histamine-induced pruritus (29). In patients with immune-related diseases, IL-31 is secreted in *situ* primarily by Th2 and Th1 lymphocytes in a subacute phase continuing during the chronic phase. IL-31 has 2 receptors: oncostatine M-receptor (OCMR) and IL-31 receptor A (IL-31-RA). After a repetitive and long lasting skin process Th2 and Th1 lymphocytes would have produced in *situ* IL-31 in a sufficient concentration that it could exert his action through 2 pathways according the model of (29). In the first cascade IL-31 directly stimulates OCMR, mainly present on keratinocytes and less present on cutaneous sensory nerve fibers. The keratinocytes and the cutaneous IL-31 stimulate peripheral sensitive nerve fiber. Once the stimulus reaches dorsal root ganglia, through the sensitive path (classic itching way) it initiates the scratching reflex (direct way). The second pathway might be activated by plasmatic IL-31. In this model the interleukin links DRG (dorsal root ganglia) to IL-31 RA by blood flow. By following this pathway IL-31 might also initiate the scratching reflex (indirect way). There is not much scientific evidence about IL-31 and its relation to itch in ACD. It was found to be expressed in skin biopsies and in one study of 20 patients with moderate to severe skin manifestations due to 3 different allergens, the IL-31 blood levels were significantly higher in patients than in controls (30). The IL-31 levels were not related to the allergen involved and did not change on the strength of the allergen involved. This could mean that the IL-31 levels are related to the itch and not to the skin damage extend.

## IL-33

Also IL-33, a proinflammatory cytokine and member of the IL-1 family seems to play a role in allergic skin diseases. The main cellular sources of IL-33 are epithelial cells and endothelial cells. IL-33 is released by cells undergoing necrosis (31). It is closely involved in Th2 immune responses. The importance of IL-33 in the pathogenesis of atopic dermatitis is considerable (32). Besides its proinflammatory effect it has also a negative effect on the skin barrier and thus reduces the protective function of the skin to pathogenic germs and allergens. Moreover, IL-33 also induces pruritus indirectly via IL-31. Due to this pruritus the AD patients are going to scratch themselves causing further damage to the skin. This means release of more IL-33, creating a scratch-pruritus cycle.

Concerning ACD TNF-α and interferon-γ which are involved in the pathogenesis of ACD as Th1 immune response could induce the expression of IL-33 in KERTr cells (a human keratinocyte cell line) (31). Expression of IL-33 can promote Th2 immune responses in keratinocytes, indicating coexistence of Th1 and Th2 immune response in ACD. Liu et al. identified IL-33, using transcriptome microarray analysis, as a key cytokine up-regulated in the inflamed skin of urushiol-challenged mice (mouse model of poison ivy contact allergy) (7). They also observed that the serum IL-33 receptor, ST2, is expressed in small to medium-sized dorsal root ganglion neurons, including neurons innervating the skin. Neutralizing antibodies against IL-33 or ST2 reduced scratching behavior and skin inflammation in urushiol-challenged mice. However, blocking IL-33/ST-2 could not completely eliminate itch-related behavior, which means that other itch pathways play a role in this model.

The same study which showed that the IL-31 levels were significantly higher in patients with ACD showed that the IL-33 serum levels were similar in patients and healthy control patients (30). Taking into consideration that ACD is a local event without systemic involvement, local IL-33 seems to function as a kind of early warning system.

## TSLP

Thymic stromal lymphopoietin (TSLP) is an epithelial-derived cytokine and a master initiator of Th2 type inflammation (33). Keratinocytes release TSLP in response to a range of stimuli including protease activation of protease-activated receptor 2 (PAR2). Both subunits of the TSLP receptor, IL7Rα and TSLPR are detected in a small subset of nociceptive neurons that do not overlap with either histamine or chloroquine-responsive neurons. TSLP can trigger itch through direct neuronal activation via the TSLP receptor.

Liu et al. could demonstrate in a study comparing mouse ACD models induced by the poison ivy allergen urushiol and the synthetic allergen oxazolone that neutralizing TSLP could significantly diminish scratching behavior in urushiol-challenged mice, suggesting a key role in the itch response of poison ivy ACD (10). In the oxazolone model, they found only minimal amounts of TSLP in the skin and none in plasma. Neutralizing TSLP did not reduce the itch behavior in the oxazolone model. In contrast, elevated skin levels of neuropeptide SP were observed in the oxazolone model, which was not the case in the urushiol model. These observations indicate that also these two models use distinct pruritogenic pathways.

## Discussion

Contact dermatitis is a growing environmental and occupational health problem. It results in high costs for health care systems and the economy due to loss of productivity (1). Moreover it has a huge impact on the quality of life of the individual patient.

Over the last years we have received new interesting insights into the pathogenesis of ACD: the identification of distinct immune pathways and the activation of these pathways by different allergens (9). Itch is a major symptom in ACD causing a relevant burden on patient's quality of life. The focus on non-histaminergic itch-sensory pathways is a big step forward for patients with chronic itch disorders as ACD. These findings about the pathogenesis of ACD and the development of itch in ACD patients create perspectives. Topical steroids are effective in the short term for most of the patients. For the difficult to treat and/or chronic cases topical calcineurin inhibitors, light therapy and systemic agents as steroids and cyclosporine are used. For the associated itch antihistamines are largely ineffective. The innovative topical or systemic drugs, targeting single specific itch mediators (anti-cytokine monoclonal antibodies as anti-IL-31, anti-IL-33 and anti-TSLP) or inhibiting multiple pruritogenic cytokines by blocking common signal transduction pathways (anti-JAKS) actually already used or under investigation in atopic dermatitis seem plausible candidates for difficult to treat patients. The discovery of the Mrgpr circuit (22, 24) and the observation of (25) about the preferential role of Mrgprb2 in ACD and associated pruritus make it a novel therapeutic target.

## Author Contributions

The author confirms being the sole contributor of this work and has approved it for publication.

## Conflict of Interest

The author declares that the research was conducted in the absence of any commercial or financial relationships that could be construed as a potential conflict of interest.

## Publisher's Note

All claims expressed in this article are solely those of the authors and do not necessarily represent those of their affiliated organizations, or those of the publisher, the editors and the reviewers. Any product that may be evaluated in this article, or claim that may be made by its manufacturer, is not guaranteed or endorsed by the publisher.
